# Characterization of proteinases from the midgut of *Rhipicephalus *(*Boophilus*) *microplus *involved in the generation of antimicrobial peptides

**DOI:** 10.1186/1756-3305-3-63

**Published:** 2010-07-27

**Authors:** Carlos E Cruz, Andréa C Fogaça, Ernesto S Nakayasu, Cláudia B Angeli, Rodrigo Belmonte, Igor C Almeida, Antônio Miranda, Maria Terêsa M Miranda, Aparecida S Tanaka, Glória R Braz, Charles S Craik, Eric Schneider, Conor R Caffrey, Sirlei Daffre

**Affiliations:** 1 Department of Parasitology, Institute of Biomedical Sciences, University of São Paulo, São Paulo, SP 05508-900, Brazil; 2 The Border Biomedical Research Center, Department of Biological Sciences, UTEP, El Paso, TX 79968, USA; 3 Department of Biophysics, Federal University of São Paulo, São Paulo, SP 04044-020, Brazil; 4 Department of Biochemistry, Institute of Chemistry, USP, São Paulo, SP 05508-900, Brazil; 5 Department of Biochemistry, Federal University of São Paulo, São Paulo, SP 04044-020, Brazil; 6 Department of Biochemistry, Institute of Chemistry, Federal University of Rio de Janeiro, Rio de Janeiro, RJ 21941-909, Brazil; 7 Department of Pharmaceutical Chemistry, University of Califormia San Francisco, San Francisco, CA 94158, USA; 8 Sandler Center for Drug Discovery, University of California San Francisco, San Francisco, CA 94158, USA

## Abstract

**Background:**

Hemoglobin is a rich source of biologically active peptides, some of which are potent antimicrobials (hemocidins). A few hemocidins have been purified from the midgut contents of ticks. Nonetheless, how antimicrobials are generated in the tick midgut and their role in immunity is still poorly understood. Here we report, for the first time, the contribution of two midgut proteinases to the generation of hemocidins.

**Results:**

An aspartic proteinase, designated BmAP, was isolated from the midgut of *Rhipicephalus *(*Boophilus*) *microplus *using three chromatographic steps. Reverse transcription-quantitative polymerase chain reaction revealed that BmAP is restricted to the midgut. The other enzyme is a previously characterized midgut cathepsin L-like cysteine proteinase designated BmCL1. Substrate specificities of native BmAP and recombinant BmCL1 were mapped using a synthetic combinatorial peptide library and bovine hemoglobin. BmCL1 preferred substrates containing non-polar residues at P2 subsite and polar residues at P1, whereas BmAP hydrolysed substrates containing non-polar amino acids at P1 and P1'.

**Conclusions:**

BmAP and BmCL1 generate hemocidins from hemoglobin alpha and beta chains *in vitro*. We postulate that hemocidins may be important for the control of tick pathogens and midgut flora.

## Background

The one-host ixodid tick *Rhipicephalus *(*Boophilus*) *microplus *is a haematophagous ectoparasite of great veterinary importance in tropical and subtropical regions, owing to its involvement in the transmission of diseases to cattle, such as babesiosis and anaplasmosis, that result in severe economic losses [1].

Besides transmission of pathogens, severe infestations with *R*. (*B*.) *microplus *cause anaemia in cattle, as a single engorged female can increase its weight by 100 to 200 times [2]. Such prodigious engorging capacity involves proteolysis of host proteins, which occurs predominantly inside acidic vesicles of digestive cells. During digestion the products of proteolysis are transferred to the haemocel whereas the resulting heme is continuously segregated into hemosomes [3-5].

In ixodid ticks, the main proteolytic activities in the midgut are acidic aspartic and cysteine proteinases; exopeptidases may also participate in the final stages of digestion [6-9]. A cathepsin D was partially purified from engorged female midguts of *R*. (*B*.) *microplus *with an optimum hemoglobinolytic activity around pH 3.0 [7]. Also, a hemoglobinolytic cathepsin L-like enzyme, named BmCL1 [GenBank:AF227957], was characterized and immunolocalized to the midgut cells of partially engorged adult female ticks [8,10].

This initial enzyme characterization suggests that *R*. (*B*.) *microplus *utilizes a digestive network of aspartic and cysteine proteinases similar to those described in other hematophagous parasites including *Ixodes ricinus *[6,11], nematodes [12] and flatworms [13,14]. In ticks, some of the identified aspartic proteinases are expressed only in the midgut and their expression levels increase during blood feeding [11]. In *R*. (*B*.) *microplus*, the contribution of other cysteine-proteinases involved in vitellin degradation has also been addressed [16,17].

Digestion of host blood proteins, particularly hemoglobin, is essential not only to supply nutrients for molting and vitellogenesis, but also to generate hemocidins that may act as a defense mechanism against microorganisms [18-20]. The first isolated hemocidin came from the midgut contents of *R*. (*B*.) *microplus*, and was named Hb 33-61. Its synthetic analogue was active against Gram-positive bacteria and fungi in micromolar concentrations [18].

The structure of the synthetic amidated peptide Hb 33-61a [21] as well as its truncated analogues [22] was elucidated by H^1^-NMR in micelles of SDS. This data suggested that the amino-terminal region of Hb 33-61a serves as an anchor to stabilize the peptide into the SDS membrane, whereas a C-terminal alpha helical portion is responsible for membrane permeabilization. Interestingly, several other hemocidins generated through proteolytic digestion *in vitro *contain a high α-helical content [23-25].

Here we report the characterization of two proteinases from *R*. (*B*.) *microplus *midgut involved in hemoglobinolysis and in the production of hemocidins. To our knowledge, this is the first report describing proteinases from a living organism that are responsible for the production of a hemocidin *in vivo*. The present report highlights the potential role of hemocidins in the control of pathogens acquired by ticks as well as the midgut flora.

## Results

### Proteinase activity in *R*. (*B*.) *microplus *midgut homogenate and digestive cell lysate

To determine which proteinase classes from the midgut homogenate and digestive cell lysate are responsible for the cleavage of hemoglobin to generate the hemocidin Hb 33-61, we used the fluorescent substrates SF 29-35 (Abz-LERMFLSQ-EDDnp) and SF 57-67 (Abz-GHGAKVAAALTQQ-EDDnp) containing the amino acid sequences 29-35 and 57-67 of the α-chain of bovine hemoglobin, and flank the N- and C-terminus of Hb 33-61, respectively, in combination with specific inhibitors.

High fluorescence was detected in midgut homogenate (Table 1A) and digestive cells (Table 1B) at pH 4.5 using SF 29-35, and this activity was inhibited only by pepstatin. At pH 7.0, only a trace of enzymatic activity was detected. Thus, we conclude that midgut-associated acidic aspartic proteinases are involved in the cleavage of SF 29-35.

**Table 1:** Specific enzyme activities in the tick midgut.

A						
			SF 29-35			

	pH 4.5				pH 7.0	
		
Enzyme activity	+ pepstatin	+ E-64		Enzyme activity	+ pepstatin	+ E-64
						
5,471 ± 238	320 ± 6.3^a^	5,295 ± 173		81 ± 25	65 ± 9.4	62 ± 7.4
						
			SF 57-67			

	pH 4.5				pH 7.0	
						
Enzyme activity	+ pepstatin	+ E-64		Enzyme activity	+ pepstatin	+ E-64
						
141 ± 18	139 ± 17	88 ± 14^a^		171 ± 21	184 ± 10	168 ± 20

						

B						

			SF 29-35			

	pH 4.5				pH 7.0	
		
Enzyme activity	+ pepstatin	+ E-64		Enzyme activity	+ pepstatin	+ E-64
						
6,072 ± 797	329 ± 27^a^	6,115 ± 206		146 ± 59	154 ± 21	109 ± 29
						
			SF 57-67			

	pH 4.5				pH 7.0	
		
Enzyme activity	+ pepstatin	+ E-64		Enzyme activity	+ pepstatin	+ E-64
						
138 ± 11	108 ± 6.6	33 ± 12^a^		319 ± 33	320 ± 20	320 ± 26

Enzyme activities (RFU × 10^3 ^× min^-1 ^× mg prot^-1^) were determined in midgut homogenates **(A) **and digest cell lysate **(B) **in the presence of pepstatin and E-64 at pH 4.5 and 7.0 using SF 29-35 and SF 57-67. Data represent mean ± SEM from three samples. a: p < 0.05.

Conversely, using SF 57-67, a low fluorescence was detected at pH 4.5 and 7.0. However, this activity was significantly inhibited by E-64 at pH 4.5 and no significant enzyme inhibition was observed in the presence of pepstatin at either pH value (Table 1, panels A and B). Therefore, we conclude that midgut acidic cysteine-proteinases are involved in the cleavage of SF 57-67 and that their specific activity was 40 times lower than that of aspartic proteinases in both midgut homogenate and digestive cells.

### Purification, kinetic analyses and cleavage specificity of BmAP

**Figure 1:**
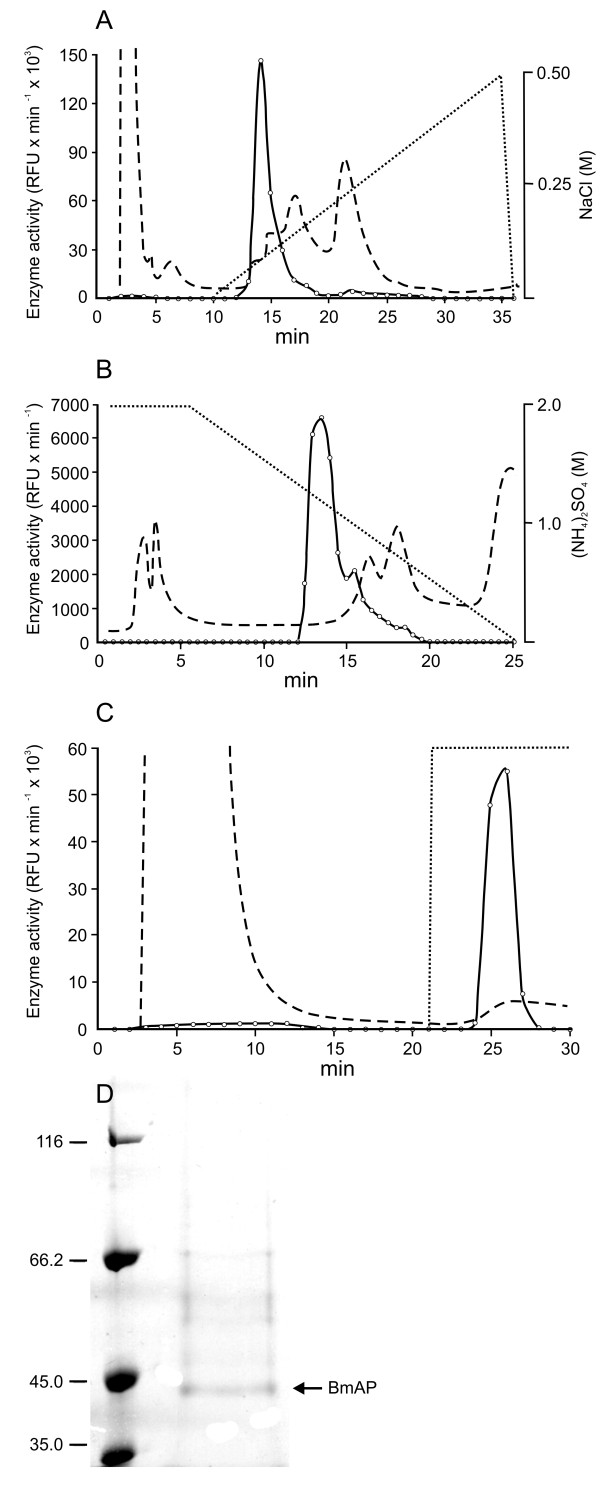
**Purification of aspartic proteinases from tick midgut homogenates**. Purification was accomplished in three chromatographic steps: anion exchange chromatography **(A)**, hydrophobic interaction chromatography **(B) **and pepstatin affinity chromatography **(C) **in FPLC system. Enzyme activity was assayed with SF 29-35 (-o-). Absorbance was monitored at 280 nm (- -). NaCl gradients (---) were developed as described in Methods. **(D) **SDS-PAGE of active fraction eluted from the pepstatin affinity chromatography (see also Additional File 1).

BmAP was purified to apparent homogeneity in three chromatographic steps. Midgut homogenate supernatant was initially resolved using a Mono Q column. The eluted fractions were screened with SF 29-35 and a single activity peak, eluted at 80 mM NaCl, was detected and inhibited by pepstatin (Figure 1A). This active fraction was collected and further resolved by hydrophobic interaction chromatography. A single activity peak was eluted at 1.2 M (NH_4_)_2_SO_4 _(Figure 1B). As a last purification step, active fractions were pooled and loaded onto a pepstatin affinity column and a single active fraction was obtained by a stepwise elution with 0.1 M Tris-HCl, 1 M NaCl, pH 8.6 (Figure 1C). Enzyme recoveries after each purification step were 67%, 30% and 21%, respectively. The purified enzyme was designated BmAP.

SDS-PAGE analysis of BmAP showed a protein band at approximately 42 kDa (Figure 1D). The protein band was excised, reduced with DTT, alkylated with iodoacetamide, and in-gel digested with trypsin. The resulting tryptic peptides were analysed by LC-MS/MS and the collected MS/MS spectra searched against the non-redundant NCBI and *R. (B.) microplus *BmiGI databases, resulting in the identification of the peptides VVFDTGSSNLWVPSSK, FDGILGLGYPR and YYTIFDR. Together, these three peptides unambiguously matched to a single aspartic proteinase sequence from the BmiGI database (contig #3, BmiGI number TC 18173) [GenBank:FJ655904].

**Figure 2:**
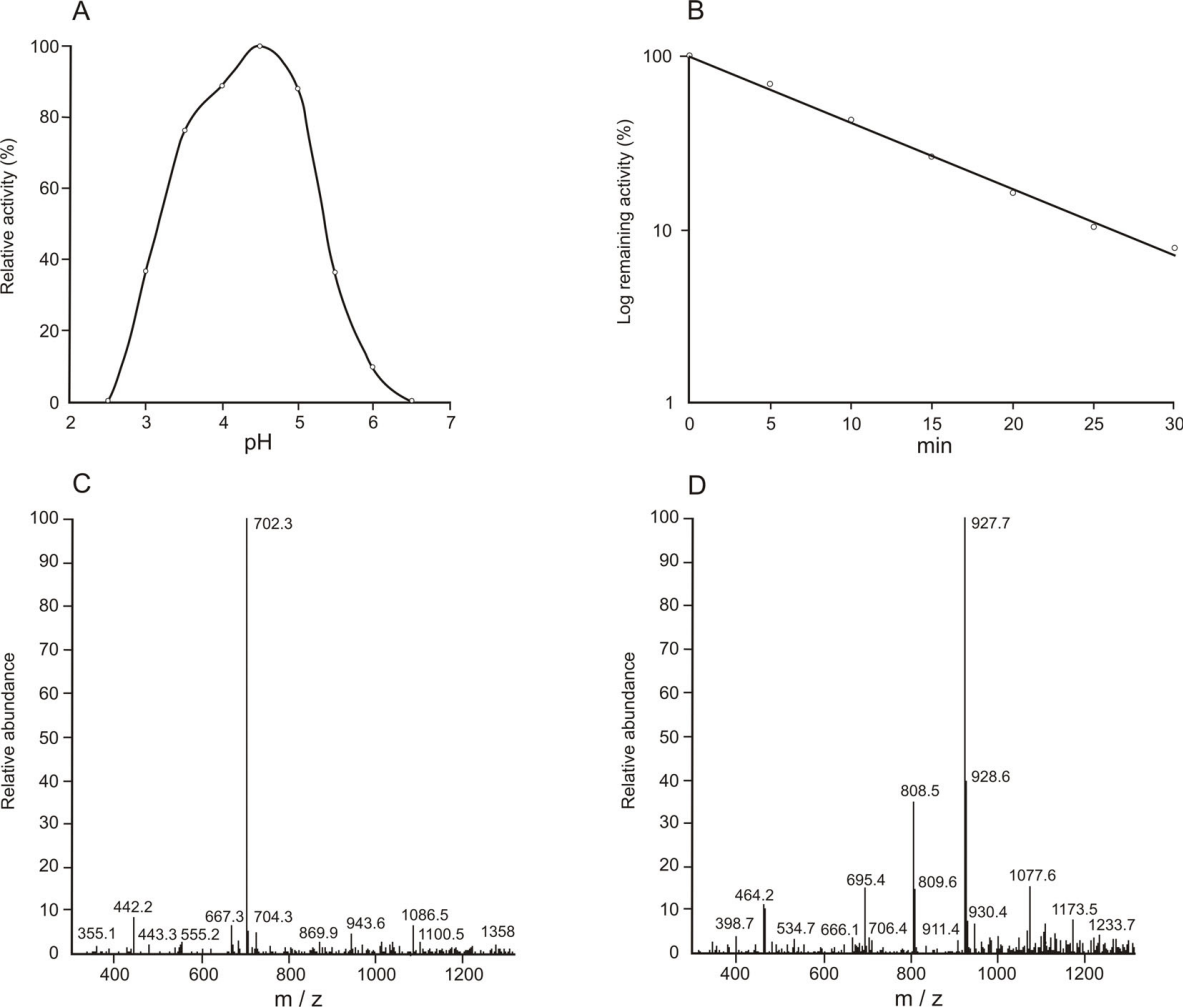
**Biochemical characterization of BmAP**. **(A) **Effect of pH on BmAP activity. Buffer used: 100 mM citrate-phosphate pH 2.5-7.5. **(B) **Thermal inactivation of BmAP at 68°C at pH 4.5. **(C) **and **(D) **Determination of the cleavage sites of SF 29-35 by BmAP using LC/MS.

After purification of BmAP, some of its kinetic properties were determined. The enzyme has an optimum activity at pH 4.5 (Figure 2A) and thermally inactivates with a half-life of 8 min at 68°C, according to apparent first-order kinetics, suggesting the presence of only one isoform in the sample (Figure 2B).

Analysis of the cleavage specificity of BmAP using SF 29-35 resulted in the detection of singly charged ions at *m/z *702.3, corresponding to the fragment FLSQ-EDDnp (theoretical *m/z *of 702.4 Da), demonstrating the cleavage of SF 29-35 at Met32/Phe33 (Figure 2C). Additionally, an ion at *m/z *927.7 was detected, corresponding to the fragment Abz-LERMFL (theoretical *m/z *of 927.5 Da), and consequently to the cleavage at Leu34/Ser35 (Figure 2D).

Chromatographic fractions eluted from the Mono Q column were also assayed with SF57-67. Two active fractions, eluted at 0.16 and 0.22 M NaCl, were inhibited only by E-64, and these fractions were active against Z-Phe-Arg-MCA but not against Z-Arg-Arg-MCA, which suggests that they contain cathepsin L-like proteinases (data not shown).

### cDNA sequencing of BmAP

**Figure 3:**
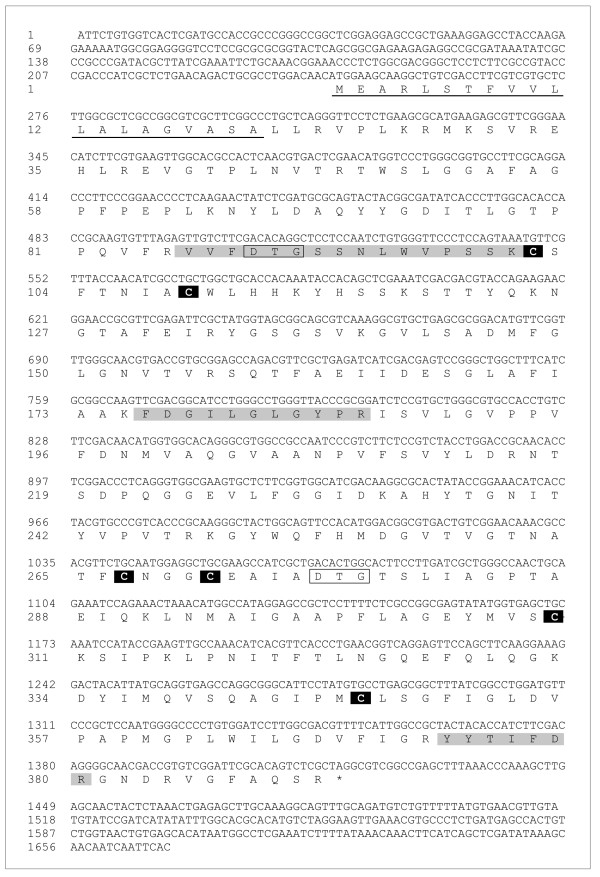
**Nucleotide and deduced amino acid sequence of *R*. (*B*.) *microplus *aspartic proteinase cDNA**. The predicted 20-aa signal peptide is underlined. The conserved residues involved in catalysis are in rectangles. The regions identified by LC-MS/MS are shaded. The cysteine residues involved in dissulfide bond formation are in black. The GenBank accession number for this sequence is FJ655904.

Using specific oligonucleotides designed from the identified contig #3, a full-length cDNA was obtained. Its nucleotide sequence encodes a protein containing 392 amino-acid residues, with a predicted molecular mass of 42.2 kDa (Figure 3). Using Signal P, the most likely signal peptide cleavage site was identified between residues 20 (Ala) and 21 (Leu) of the preprotein, resulting in a 372 amino-acid mature polypeptide with a molecular mass of 40.2 kDa and a theoretical isoelectric point of 8.2.

**Figure 4:**
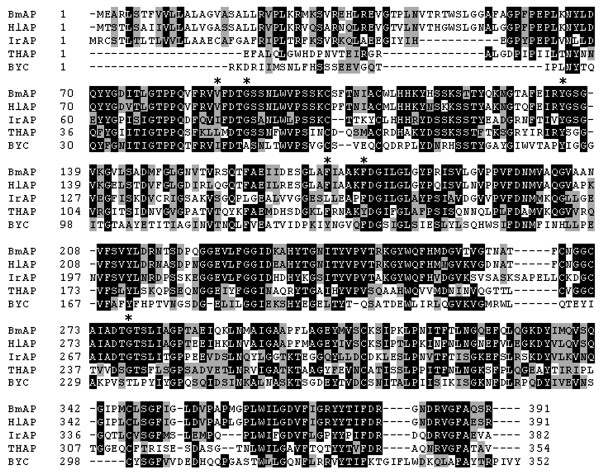
**Amino acid sequence alignment of aspartic proteinases**. BmAP, *Rhipicephalus *(*B*.) *microplus *aspartic proteinase [GenBank:FJ655904]; HlAP, *Haemaphysalis longicornis *aspartic proteinase [GenBank:BAE53722]; IrAP, *Ixodes ricinus *aspartic proteinase [GenBank:EF428204]; THAP, tick heme-binding aspartic proteinase [GenBank:AF286865]; BYC, *Boophilus *Yolk pro-Cathepsin [GenBank:AY966003]. The catalytic residues are indicated with asterisks.

The primary structure of the deduced amino acid sequence shows the presence of two catalytic triads at Asp^89^-Thr^90^-Gly^91 ^and Asp^276^-Thr^277^-Gly^278 ^(Figure 3). A SwissModel protein structure homology modelling indicates the presence of three internal disulfide bridges at Cys^102-109^, Cys^267-271 ^and Cys^310-347^. Amino acid sequence alignment using Clustal × showed an identity of 82% and 52% with cathepsins D from *Haemaphysalis longicornis *[GenBank:AB218595] and *Ixodes ricinus *[GenBank:EF428204], respectively, and 44% and 30% identity with the *R*. (*B*.) *microplus *aspartic proteinases THAP [GenBank:AF286865] and BYC [GenBank:AY966003], respectively (Figure 4).

### Determination of expression levels of BmAP and BmCL1

Quantitative RT-PCR profiling of mRNA levels in the midgut, salivary glands and ovaries of fully engorged female ticks revealed that BmAP is only expressed in the midgut, showing a ΔC_t _value of 6.99 ± 0.68. Likewise, BmCL1 is only expressed in the midgut of adult females, with a ΔC_t _value of 1.91 ± 0.25.

### Expression of BmCL1 and determination of its substrate specificity

**Figure 5:**
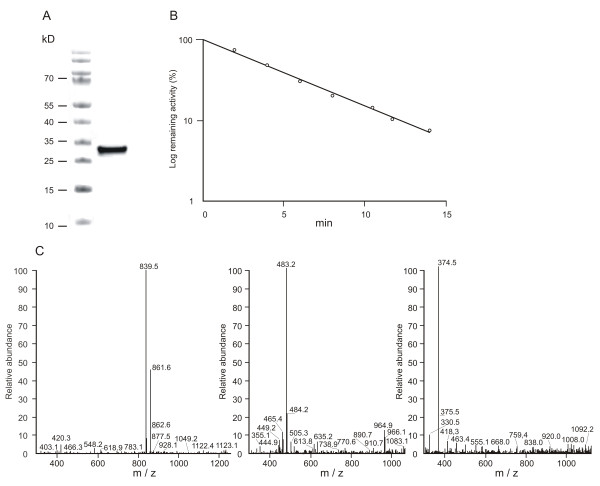
**Biochemical properties of recombinant BmCL1 expressed in *Pichia pastoris***. **(A) **SDS-PAGE of BmCL1 purified on ion exchange chromatography, as described in Methods. **(B) **Thermal inactivation at 68°C at pH 4.5. **(C) **Determination of the cleavage sites of SF 57-67 by BmCL1 using LC/MS.

The recombinant cathepsin-L proteinase BmCL1 [GenBank:AF227957] was expressed in *Pichia pastoris *as an apparently mature, fully active enzyme with a molecular mass of approximately 30 kDa, as determined by SDS-PAGE (Figure 5A). In a thermal inactivation assay, the recombinant enzyme showed a linear decrease in activity, with a half-life of 3.7 min, confirming the presence of only one isoform purified from *Pichia *culture medium (Figure 5B).

LC/MS analysis of the products derived from the hydrolysis of SF 57-67 by BmCL1 detected singly charged ions at *m/z *839.5, corresponding to the mass of the peptide AALTQQ-EDDnp (theoretical *m/z *of 839.5) and at *m/z *483.2, corresponding to the peptide QQ-EDDnp (theoretical *m/z *of 483.3 Da), demonstrating cleavage of this substrate at Ala63/Ala64 and Thr67/Gln68, respectively (Figure 5C). The fragment AALT, formed by the simultaneous cleavage of SF 57-67 at Ala63/Ala64 and Thr67/Gln68, was also detected at *m/z *374.5 (theoretical *m/z *of 375.4 Da) (Figure 5C). Interestingly, LC/MS analysis after hydrolysis of SF 57-67 by the cathepsin L-like proteinases partially resolved by anion-exchange chromatography or midgut homogenate at pH 4.5 resulted in the detection of the same ions at *m/z *483.2 and 839.5 (data not shown), which correspond to the cleavage sites determined for BmCL1.

**Figure 6:**
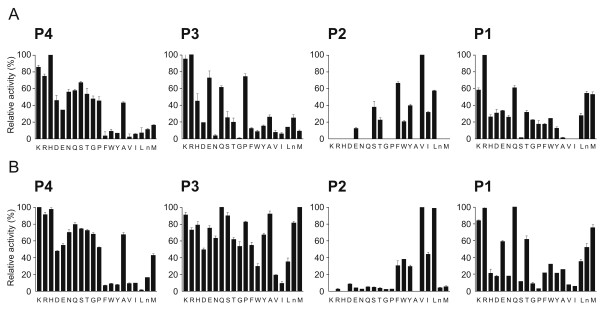
**Subsite specificity of recombinant BmCL1 using a tetrapeptide library**. A P1-P4 positional scanning synthetic combinatorial library (PS-SCL) was used to determine specificities at pH 4.5 **(A) **and pH 5.5 **(B)**. Activities are represented as the percentage of the maximum activity for each subsite position. Data represent means ± SEM of three runs.

To fully understand the substrate specificity of recombinant BmCL1, assays were performed with a positional scanning synthetic combinatorial library (PS-SCL). Cleavage specificity profiling at pH 4.5 using the PS-SCL revealed that BmCL1 preferentially cleaves substrates containing polar side chains at position P1 (Figure 6, panel A). As expected for papain-like cysteine proteinases [26], Arg was preferred at P1, but other residues could also be accommodated at this position, including Gln, Lys and Met. At P2, BmCL1 preferred aliphatic residues (mainly Val and Leu) and aromatic residues (mainly Phe and Tyr) (Figure 6, panel A). Moreover, the enzyme preferred polar residues at P3 and P4 (Figure 6, panel A). Similar specificities at P1-P4 were observed at pH 5.5, with slightly higher hydrolysis rates being detected with Leu over Phe at P2 (Figure 6, panel B). At pH 5.5, BmCL1 could also hydrolyse peptides containing Thr and Glu at P1 (Figure 6, panel B). This preference for Val/Leu at P2 supports the observed cleavage of SF 57-67 at Ala63/Ala64 and Thr67/Gln68.

### Generation of hemoglobin-derived peptides by BmAp and BmCL1

**Figure 7:**
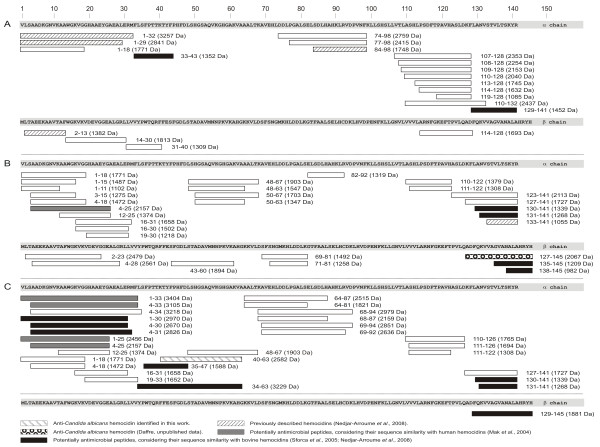
**Peptides generated by acid hemoglobinolysis**. Peptides were identified by LC-MS/MS after hemoglobinolysis with native BmAP **(A)**, BmCL1 **(B) **or both enzymes **(C)**. Digestion was performed in 100 mM citrate-phosphate buffer pH 4.5 at 37°C for up to 4 h, as described in Methods.

Figure 7 summarizes the cleavage specificities of native BmAP, recombinant BmCL1 and both enzymes against either alpha or beta subunit of bovine hemoglobin *in vitro*. Mass spectrometric analysis of the peptides generated by BmAP identified 20 peptide sequences, with molecular masses ranging from 1085 Da to 3257 Da; sixteen were derived from the α subunit and four from the β subunit of bovine hemoglobin (Figure 7, panel A). BmAP preferentially hydrolysed hemoglobin at sites containing non-polar amino acids at P1 (81% of the cleavage sites), of which the most prevalent residues were Leu and Phe (62%) and predominantly at sites containing hydrophobic residues at P1' (65%). Cleavage sites were found in alpha helical and random coil regions of hemoglobin and resulted in the generation of several peptides previously shown to be antimicrobial [25], such as α1-29, α1-32, α84-98 and β1-13 (Figure 7, panel A) as well as potential antimicrobials, such as α33-43 and α129-141, due to their sequence similarity with the antimicrobials α34-46 and α133-141, respectively [25].

Mass spectrometric analysis of the peptides generated by *in vitro *hemoglobinolysis using recombinant BmCL1 identified 30 peptide sequences (Figure 7, panel B). Considering all the cleavage sites identified, 63% of them were composed of polar residues at P1. In contrast, 81% of them had apolar residues at P2, with a preference for Leu and Val. No apparent substrate preference was found at the P1' or P2' subsites. Cleavage sites were found in alpha helical and random coil regions of hemoglobin and resulted in the generation of several peptides, two of which were previously shown to be antimicrobial: α133-141 [25] and β127-145 (Daffre, unpublished data) (Figure 7, panel B). In addition, peptides α4-25, α130-141, α131-141, β135-145 and β138-145 are potential antimicrobials, considering their sequence similarity with known antimicrobials [25] (Figure 7, panel B). Likewise, hemoglobinolysis with both proteinases allowed for the identification of 30 peptides (Figure 7, panel C), two of which were previously reported as human hemocidins (α1-25 and α1-33) [27] and 10 of them as potential antimicrobials [25,27] (Figure 7, panel C).

Although the hemocidin Hb 33-61 previously isolated from *R*. (*B*.) *microplus *midgut [18] was not generated by *in vitro *hemoglobinolysis, cleavage at Met32/Phe33 was shown to occur through the activity of BmAP, as observed by the formation of α1-32 and α33-43 (Figure 7, panel A). As expected, cleavage at Lys61/Val62 to generate Hb 33-61 was not observed in hemoglobin, considering the specificity of BmCL1 (Figure 6, panels A and B). However, the peptides α48-63 and α50-63, produced by the activity of BmCL1, and α40-63, produced by the activity of both enzymes, were identified in the hydrolysate (Figure 7, panels B and C), demonstrating cleavages related with Hb 33-61.

Considering the structure/activity studies of Hb 33-61 and its truncated analogues Hb 33-52, Hb 40-61 and Hb 48-61 [21,22], we decided to compare the anticandidal activity of the peptide containing the additional residues Val62 and Ala63 (Hb 40-63) with these peptides previously characterized. To accomplish that, Hb40-63 was synthesized by the solid-phase methodology, purified by RP-HPLC and characterized by LC-MS. Its MIC against *Candida albicans *was 3.12-6.25 μM, which is 4-fold more active than Hb 33-61 and its analogue Hb 40-61 [22].

## Discussion

Hydrolysis of the Abz-containing fluorogenic substrates in the presence of midgut homogenate and digestive cell lysate showed that aspartic proteinases are involved in the cleavage of SF 29-35, whereas cysteine proteinases hydrolyse SF 57-67. Considering that specific activities in midgut homogenate were very similar to the activities in digestive cells (Table 1), hemoglobin digestion seems to occur intracellularly, which is consistent with previous data [5].

In a previous work, a *R*. (*B*.) *microplus *midgut aspartic proteinase was partially resolved by ion exchange chromatography [7]. Here we describe the purification, cloning and cleavage specificity of an aspartic proteinase and confirmed its identity as a cathepsin D. By using three chromatographic steps and performing a thermal inactivation assay, we found only one aspartic proteinase isoform in the tick midgut. Analysis by RT-qPCR showed that BmAP is only expressed in the midgut, but at present we are uncertain whether its expression increases during blood feeding, as observed for other tick aspartic proteinases [28].

A few aspartic proteinases have been characterized from the eggs of *R*. (*B*.) *microplus*. *Tick heme-binding aspartic proteinase *(THAP) is a cathepsin-D like proteinase involved in vitellin digestion, and contains conserved cysteine residues and two typical active sites [29]. Another tick aspartic proteinase named BYC (*Boophilus yolk cathepsin*), in contrast, lacks the second Asp catalytic residue. Like BmAP, BYC prefers substrates containing hydrophobic residues at the P1 and P1' subsites, which is in accordance with the specificity of most aspartic proteinases reported, including those from humans, the schistosome bloodfluke and ixodid ticks [6,12,30-32]. Importantly, we show that BmAP is responsible for the generation of antimicrobial peptides, such as α1-32 (Figure 7, panel A), which was previously reported as being active against a broad range of microorganisms [25].

The second proteinase under investigation, BmCL1, was previously shown to be immunolocalized in midgut cells of partially engorged adult females, corroborating its involvement in hemoglobinolysis [8]. It was also verified that BmCL1 is only expressed in the midgut [8], and this data was here confirmed by RT-qPCR. In other tick species, cysteine proteinases were also reported to contribute to hemoglobinolysis at acidic pH, and to be upregulated by blood feeding [6,15]. Interestingly, a longipain has been characterized in *Haemaphysalis longicornis *which may function in the regulation of their vectorial capacity for *Babesia *parasites [33].

Most variations in papain-like proteases occur at residues 67 and 205 at the S2 subsite of the active site, and these residues may reflect differences in substrate specificity [34]. Like human cathepsin L, BmCL1 possesses a Leu in position 67, but unlike the human orthologue, it possesses a Gln in residue 205 instead of Ala [10], which may explain the lower specificity for bulky aromatic residues compared with other cathepsin L proteinases at pH 5.5 [26]. However, cathepsin L proteinases possessing other residues at position 205, such as Val and Ser, have also been reported [11,35]. Thus, additional studies will be essential to elucidate the character of the S2 pocket of BmCL1.

BmCL1 was successfully expressed in its active form, which allowed for the characterization of its substrate specificity. PS-SCL mapping revealed that BmCL1 prefers substrates containing polar residues at P1, as previously reported for other cysteine proteinases from trematode flukes and mammals [26,34,36]. BmCL1 showed a broad specificity at positions P3 and P4, which is in accordance with the specificities of other cathepsins L using similar libraries [26,34]. For P2, PS-SCL mapping demonstrated BmCL1's preference for substrates containing nonpolar residues (mainly Val, Leu, Phe and Tyr), similarly to what was reported for human cathepsin L but, unlike cathepsin K, BmCL1 does not accommodate Pro at P2 and, unlike cathepsin B, does not accept Arg at P2 [26,37,38].

Also, PS-SCL data suggest that BmCL1 can process hemoglobin at multiple sites. In fact, Val, Phe and Leu were the most prevalent residues at P2 using both PS-SCL and hemoglobin. With the PS-SCL, BmCL1 preferred Val over Phe over Leu at pH 4.5, whereas in hemoglobin it preferred Leu over Val over Phe at the same pH. Likewise, a preference for Leu/Val was observed for *Plasmodium *falcipain-2 and falcipain-3 using a similar P1-P4 library [39].

The PS-SCL specificity also showed that BmCL1 does not hydrolyse substrates containing Ala at P2, which corresponds with the proteinase not hydrolyzing alpha globin at Lys61/Val62 to generate the C-terminus of Hb 33-61 (which has Ala at P2). Moreover, when SF 57-67 was employed as substrate, cleavage at Ala63/Ala64 (with Val at P2) was detected (Figure 5C), and the same cleavage pattern occurred when using midgut extract or the cysteine proteinases partially purified from the midgut (data not shown). Thus we hypothesize that cysteine proteinases are indirectly involved in the generation of Hb 33-61, and midgut exopeptidases may further degrade peptide fragments having Ala 63 at the C-terminus providing Hb 33-61 *in vivo*.

Hydrolysis of hemoglobin by either or both BmAP and BmCL1 resulted in the generation of several peptides (summarized in Figure 7), some of which have primary structures similar to known antimicrobials, suggesting an antimicrobial activity. Interestingly, hydrolysis of hemoglobin by BmAP and BmCL1 resulted in the generation of hemocidin Hb 40-63. The synthetic form of Hb 40-63 presented a MIC of 3.12-6.25 μM against *Candida albicans*, which is four times more potent than Hb 33-61 and the truncated analogue Hb 40-61, suggesting that the presence of Val62 and Ala63 at the carboxy-terminus induces a more favorable peptide interaction with the fungal plasma membrane. Also, the peptide Hb 34-63 identified in this hydrolysate (Figure 7, panel C), very likely possesses antifungal activity, considering it contains the same structural elements of Hb 33-61 [21].

Similarly to the proteinase classes identified in the midgut of *R*. (*B*.) *microplus*, a network of aspartic and cysteine proteinases has been described in the midgut of *Ixodes ricinus*, and this network is induced upon blood feeding and degrades hemoglobin at acidid conditions [6]. In that work, a peptidase-specific cleavage map was obtained using hemoglobin as substrate, demonstrating that aspartic proteinases, supported by cathepsin L and legumain, are responsible for the primary cleavage of hemoglobin, whereas cathepsins B, L and C are involved in the generation of smaller peptides [6]. Interestingly, *Ixodes *aspartic proteinases showed preference for hydrophobic residues at the P1 and P1' subsites (Leu and Phe at P1), whereas cysteine proteinases preferred polar residues at P1 [6], similarly to the specificity observed for BmAP and BmCL1.

Endogenously generated hemocidins may help control host-acquired pathogens as well as tick midgut flora. In agreement with this hypothesis, our group has identified the hemocidin Hb 98-114, which was confirmed to be endogenously generated in the midgut, and its primary sequence corroborates the specificity data presented herein (Daffre, unpublished data). The characterization of this biologically active peptide is currently underway and its activity is being tested against natural pathogens of this tick species.

## Conclusion

During blood feeding, ticks may acquire several pathogens from their host and become efficient vectors of a number of disease-causing organisms, such as *Anaplasma marginale *[40,41]. Considering that the midgut is the primary interface of the tick-pathogen interaction, this organ needs to have an efficient innate defense mechanism in order to control pathogens as well as midgut flora. The immune responses of midgut epithelial cells to parasite invasion are well-known in other hematophagous vectors, e.g. mosquitoes [42], but little information is available for ticks [43,44]. A major component of immunity in the tick midgut may include antimicrobial fragments generated by endogenous proteolytic activity [18-20].

We conclude that the enzyme network present in the tick midgut involves aspartic and cysteine proteinases, as shown in other parasites [6,11,14]. Additionally, based on the complementarity of specificity of BmAP and BmCL1 at P1, these proteinases may act in a coordinate fashion to digest hemoglobin and generate hemocidins in the tick midgut.

## Methods

### Animals

A *Rhipicephalus *(*Boophilus*) *microplus *tick colony (Porto Alegre strain, *Babesia *spp.-free) was reared on calves maintained at the Center of Biotechnology, Federal University of Rio Grande do Sul, Porto Alegre, RS, Brazil. Host-detached fully engorged females were collected and maintained at 28°C and 80% relative humidity in a BOD incubator (Fanem). Tick rearing followed institutional guidelines and was approved by the Ethics Committee of the Federal University of Rio Grande do Sul.

### Preparation of midgut homogenates and digestive cell lysate

Fully engorged female ticks were dissected in cold phosphate buffered saline (PBS, 0.14 M NaCl, 2.7 mM KCl, 10 mM Na_2_HPO_4_, 1.8 mM KH_2_PO_4_, pH 7.4). One hundred whole midguts were homogenized in a *Potter *tissue homogenizer in cold PBS and centrifuged at 10,000 *g *for 10 min at 4°C. The resulting supernatant was pooled and stored frozen. Methyl methanethiosulfonate (MMTS - Pierce) 1 mM was added to the homogenates to avoid cysteine-proteinase autolysis. Digestive cells were harvested from dissected whole midguts as previously described [5].

### Enzyme purification

Supernatant from the homogenate of 10 midguts was submitted to anion exchange chromatography on a Mono Q column (GE Healthcare). The column was equilibrated with 50 mM Tris-HCl, pH 7.6 and elution was accomplished with a linear gradient from 0 to 0.5 M NaCl over 25 min in the same buffer. All eluted fractions were assayed with the fluorogenic substrates SF 29-35 and SF 57-67 as described below. Inhibition of enzyme activity was screened with pepstatin and E-64. Active fractions were pooled and further resolved through hydrophobic interaction chromatography in a Resource Phenyl column (GE Healthcare) previously equilibrated with 50 mM Tris-HCl, pH 7.6 containing 2 M (NH_4_)_2_SO_4_. Proteins were eluted with a linear gradient of (NH_4_)_2_SO_4 _from 2 to 0 M over 20 min. The active eluate was submitted to an affinity chromatography in an immobilized-pepstatin column (Pierce) previously equilibrated with 20 mM sodium acetate, pH 5.3 containing 1 M NaCl, followed by a stepwise elution with 100 mM Tris-HCl, pH 8.6 containing 1 M NaCl. In all chromatographic steps protein absorbance was monitored at 280 nm using a LCC 500-Plus FPLC System (GE Healthcare).

### Sodium dodecyl sulphate polyacrylamide gel electrophoresis (SDS-PAGE)

Protein samples were mixed with loading buffer and resolved by SDS-PAGE 8% or 4-20% Tris-glycine Criterion gels (Bio-Rad) [45]. Proteins were stained with Coomassie Blue R-250 for at least 6 hours.

### Enzyme assays

Enzyme assays were performed using intramolecular quenched fluorogenic peptides containing *ortho*-amino benzoic acid (Abz) at the N-terminal and ethylene diamine-2-4-dinitrophenyl (EDDnp) attached to a glutamine residue at the C-terminal, and were synthesized, purified and characterized following procedures described previously [46]. These substrates, named SF 29-35 (Abz-LERMFLSQ-EDDnp) and SF 57-67 (Abz-GHGAKVAAALTQQ-EDDnp), contain the amino acid sequences 29-35 and 57-67 of the α-chain of bovine hemoglobin, and flank the N- and C-terminus of the hemocidin Hb 33-61, respectively [18]. Aspartic proteinases were assayed in 100 mM sodium citrate buffer, pH 4.5 with or without 10 μM pepstatin. Recombinant cysteine proteinase, as well as midgut homogenate cysteine proteinases eluted from the first purification step, were pre-activated at 37°C for 15 min in 100 mM sodium citrate buffer, 100 mM NaCl, 10 mM DTT and 3 mM EDTA, pH 3.5 and were assayed with the fluorogenic substrates SF 29-35 and SF 57-67 or the 7-amido-4-methylcoumarin (MCA)-containing peptides Z-Phe-Arg-MCA and Z-Arg-Arg-MCA, in 100 mM sodium citrate buffer pH 4.5, with or without 10 μM E-64. All assays were performed in triplicate, at 30°C and with a final concentration of 10 μM of each substrate. Protease inhibitors and MCA-containing substrates were purchased from Sigma.

Initial rates of hydrolysis were measured by monitoring the release of the fluorogenic groups Abz (emission-excitation: 330-420 nm) or MCA (emission-excitation: 355-460 nm) and quantitated as Relative Fluorescence Units/min (RFU/min) in a Fluoroskan Ascent FL fluorimeter (Labsystems). No correction in fluorescence baseline was necessary because midgut preparations yielded no increase in fluorescence in the absence of substrates.

For the determination of enzyme specific activities, protein was quantitated using bovine serum albumin as standard, as previously described [47]. Data are expressed as mean ± standard error of the mean (SEM). Differences were analyzed statistically with Student's *t *test and considered significant when p ≤ 0.05.

Thermal inactivation was accomplished by incubating samples in 100 mM sodium citrate pH 4.5, followed by the determination of remaining enzyme activities at different times. Inactivation constants (K_1_, s^-1^) and remaining activities (%) were determined as previously reported [48]. The pH titration curve of purified aspartic proteinase was determined in 100 mM phosphate citrate buffer at pH 2.5-7.5.

The cleavage sites of SF 29-35 and SF 57-67 were determined by nano-HPLC/MS, after substrate hydrolysis in 50 mM sodium citrate, pH 4.5 at 37°C for 18 h.

### Mass spectrometry analyses

Products derived from the hydrolysis of SF 29-35 and SF 57-67 were desalted using C_18 _reverse phase tips (ZipTip, Millipore) and loaded onto a fused silica capillary column (0.1 × 150 mm, Polymicro) packed with Vydac C_18 _(10-15 μm, 300 Å) beads and coupled to a nano-HPLC system (Ultimate model, Dionex). Peptides were eluted with a linear gradient from 5% to 56% acetonitrile in 0.2% formic acid over 60 min and directly analysed in a LCQ™ Duo mass spectrometer (Thermo Scientific), according to a previously reported procedure [49].

For sequencing of proteins separated by SDS-PAGE, samples were 'in-gel' digested according to a published procedure [50]. Tryptic fragments were desalted in Zip-Tip and analysed by nano-HPLC-MS/MS through a 120-min gradient from 5% to 56% acetonitrile in 0.2% formic acid. MS/MS analyses were performed with Bioworks Browser version 3.3 (Thermo Scientific). Peptides were identified with Sequest^® ^algorithm and validated by considering a ΔCn ≥ 0.05 and an XCorr ≥ 1.5, 2.0 and 2.5 for singly-, doubly- and triply-charged peptides, respectively. MS/MS spectra were correlated with a NCBI non-redundant database and with a *Rhipicephalus (B.) microplus *normalized cDNA library (BmiGI database - http://www.tigr.org) [51].

Peptides derived from *in vitro *hemoglobinolysis were desalted in Zip-Tip and sequenced by nano-HPLC-MS/MS using an LCQ™ Duo mass spectrometer. Peptides were analysed against a bovine protein database using Sequest and validated by considering a ΔCn ≥ 0.085 and an XCorr ≥ 1.8, 2.5 and 3.5 for singly-, doubly- and triply-charged peptides, respectively.

### Molecular cloning and quantification of transcript levels of midgut proteinases

For RNA extraction, midguts, ovaries and salivary glands were transferred to a sterile tube containing Trizol (Invitrogen) following manufacturer's instructions. One microgram of total RNA was used as template for reverse transcription (RT) using the *SMART RACE cDNA Amplification Kit *(Clontech). A cDNA encoding an aspartic proteinase was amplified from midgut cDNA using the specific oligonucleotides 5'-CTCGAGATGGAAGCAAGGCTGTCGAC-3' (sense) and 5'-GGATTCGCACAGTCTCGCTAGGAATTC-3' (antisense). These oligonucleotides were designed based on the cDNA sequence of an aspartic proteinase obtained from the BmiGI database. Briefly, 26 cDNA sequences coding for aspartic proteinases were assembled into five contigs and one singleton using the sequence assembly program *cap3 *with the default parameters provided by the server [52]. Contig #3 (BmiGI number TC 18173), which is comprised of 8 EST sequences, was used for primer design.

PCR reactions were performed with Taq DNA polymerase (Invitrogen) using 35 cycles of 1 min at 94°C, 1 min at 53°C and 1 min at 72°C. The unique amplicon obtained was cloned into *pGEM-T Easy Vector System I *(Promega) and sequenced using the *Big Dye Terminator Cycle Sequencing kit *(Applied Biosystems) in an ABI Prism 310 Automated Sequencer (Applied Biosytems). The nucleotide sequence was subjected to similarity searches in a NCBI non-redundant database using BLAST and multiple alignments were done using Clustal × 2.0 [53]. Secondary structure was predicted using Swiss-Model Workspace [54]. Signal peptide cleavage was identified with SignalP 3.0 [55]. The nucleotide sequence corresponding to the full-length open reading frame of this proteinase is deposited in GenBank under accession number FJ655904.

Aspartic proteinase and cysteine proteinase mRNA levels were determined by RT-qPCR in salivary glands, ovaries and midguts of ticks. Fourty nanograms of cDNA were subjected to RT-qPCR using the following oligonucleotides: i. aspartic proteinase, sense (5'-ATATCACCCTTGGCACACC-3') and antisense (5'-GAGATTCGCTATGGTAGCGG-3'); ii. cysteine proteinase, sense (5'-CTGGAGGGACAGCATTTTCT-3') and antisense (5'-ATGGTTGTGAAGGTGGTCTC-3'); and iii. 40 S ribosomal protein S3A (gi156026300), sense (5'-GGACGACCGATGGCTACCT-3') and antisense (5'-TGAGTTGATTGGCGCACTTCT-3') Amplifications were performed using *Script One-Step RT-PCR kit with SYBR Green *(Bio-Rad), in iQ5 Thermal Cycler equipment (Bio-Rad). Amplification levels were normalized against the 40 S ribosomal protein S3A using the ΔC_t _method [56]. Experiments were done in triplicate and data are represented as mean ± SEM.

### Recombinant expression of BmCL1

The DNA fragment encoding the gene of the cathepsin L BmCL1 [GenBank:AF227957] was amplified by PCR, using as template the pMAL-p expression vector containing the BmCL1 sequence [10]. The BmCL1 DNA was cloned into pPIC9 vector, and the protein was expressed in the methylotrophic yeast *Pichia pastoris *and purified as previously reported by others [57].

### Determination of P1-P4 specificity using a synthetic combinatorial library

Substrate specificities were determined using a complete diverse positional scanning synthetic combinatorial library (PS-SCL). Each library contained 160,000 different tetrapeptide sequences conjugated with the 7-amino-4-carbamoylmethylcoumarin (ACC) fluorophore. Synthesis of this library has been described previously [26]. One-microliter aliquots from each of the 20 sublibraries of the P1-, P2-, P3- and P4- libraries were added to the wells of a 96-well Microfluor-1 U-bottom plate (Dynex Technologies). Assays were carried out in 100 mM phosphate citrate, 10 mM DTT, 1 mM EDTA and 1% DMSO, at pH 4.5 and pH 5.5. Initial rates of hydrolysis were continuously monitored by the release of ACC using a SpectraMax Gemini fluorescence spectrometer (Molecular Devices) with excitation and emission at 380 and 460 nm, respectively, and cutoff at 435 nm. Cysteine proteinases were pre-activated and assayed as described above.

### *In vitro *hemoglobinolysis and cleavage site identification

Bovine hemoglobin (Sigma) was incubated with aspartic proteinase (BmAP) and/or cysteine proteinase (BmCL1) in 100 mM phosphate citrate buffer, pH 4.5 at 37°C for up to 4 h. Aliquots of the hydrolysate were collected at 15 min (cysteine proteinase digestion) and at 120 min (aspartic proteinase digestion) and peptides were sequenced by LC-MS/MS as described above.

### Peptide synthesis

The peptide Hb 40-63 was synthesized, purified and characterized as previously described [22].

### Anticandidal assay

Anticandidal activity was monitored against *Candida albicans *(strain MDM8) using a liquid growth inhibition assay as described previously [22]. The MIC value was recorded as the range between the highest concentration of the peptide at which yeast growth was observed and the lowest concentration that caused 100% inhibition of yeast growth. The peptide concentrations tested were in the range of 0.78-100 μM.

## Abbreviations

ABZ: *ortho*-amino benzoic acid; DTT: dithiothreitol; E-64: *trans*-epoxysuccinyl-L-leucylamido (4-guanidino) butane; EDTA: ethylenediamine tetra-acid acid; MCA: 7-amido-4-methylcoumarin; MIC: minimum inhibitory concentration; MS/MS: tandem mass spectrometry; NMR: nuclear magnetic resonance; PCR: polymerase chain reaction; PS-SCL: positional scanning synthetic combinatorial library; SDS: sodium dodecyl sulphate; RT-qPCR: real-time polymerase chain reaction;

## Competing interests

The authors declare that they have no competing interests.

## Authors' contributions

CEC and SD designed the study and prepared the manuscript. CEC performed most of the experimental work. ACF helped perform the qPCR experiments. CBA, ICA and ESN helped perform the MS/MS experiments and analyse sequencing data. AM synthesized and purified the Abz-containing fluorogenic substrates. MTMM synthesized and purified Hb 40-63. AST expressed BmCL1 in *Pichia pastoris*. GRB carried out all the sequence annotation and contig assembly. CSC, ES and CRC helped design and perform the PS-SCL experiments. RB helped analyze MS/MS data. All authors read and approved the final manuscript.

## Supplementary Material

Additional file 1 **MS/MS spectra of the aspartic proteinase amino acid sequence**. LC-MS/MS data were searched against a non-redundant NCBI database, and the amino acid sequences YYTIFDR **(A)**, VVFDTGSSNLWVPSSK **(B) **and FDGILGLGYPR **(C) **were identified by Sequest and validated using the parameters described in Methods. Peptide probabilities, DCn values and Xcorr values are given for each peptide spectra. Click here for file
