# Supplementary figures and images for: Characterization of proteinases from the midgut of Rhipicephalus (Boophilus) microplus involved in the generation of antimicrobial peptides
Source: Parasit Vectors. 2010 Jul 27;3:63. doi: 10.1186/1756-3305-3-63 (PMC2921360; doi:10.1186/1756-3305-3-63)

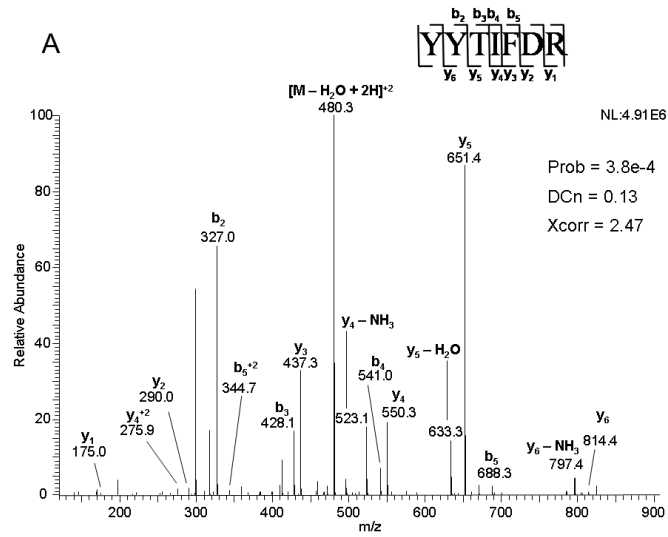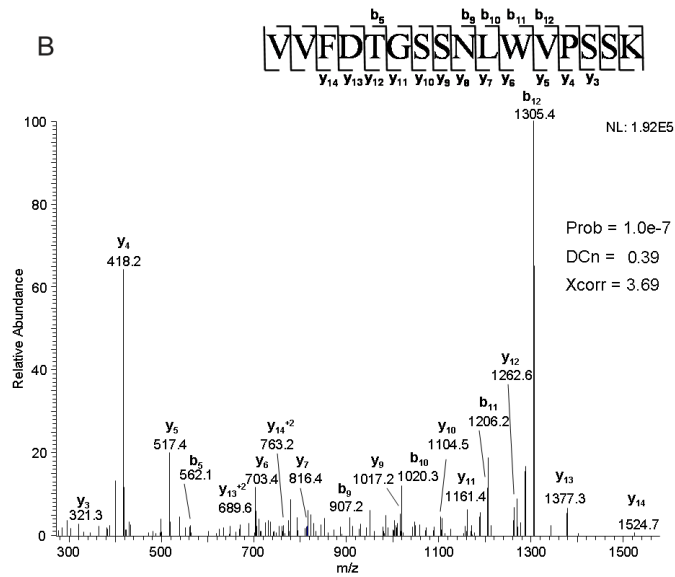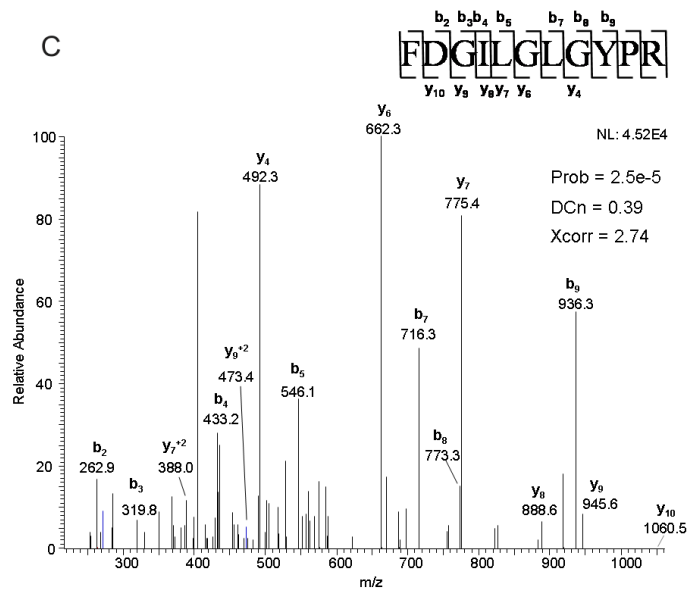

Supplement: Additional file 1 — MS/MS spectra of the aspartic proteinase amino acid sequence. LC-MS/MS data were searched against a non-redundant NCBI database, and the amino acid sequences YYTIFDR (A), VVFDTGSSNLWVPSSK (B) and FDGILGLGYPR (C) were identified by Sequest and validated using the parameters described in Methods. Peptide probabilities, DCn values and Xcorr values are given for each peptide spectra. [file 1756-3305-3-63-S1.PDF]
